# Prenylated apigenin derivatives from *Cannabis sativa* L*.*: isolation, biosynthesis, and anti-inflammatory properties

**DOI:** 10.1186/s42238-026-00438-4

**Published:** 2026-04-16

**Authors:** Ulli K. C. Bodnar, Jackson J. Villemaire-McCutcheon, Kelly F. Boddington, Eric Soubeyrand, M. Sameer Al-Abdul-Wahid, Hannah N. Robeson, Jasmin Lalonde, José A. Casaretto, Tariq A. Akhtar

**Affiliations:** 1 Department of Molecular and Cellular Biology, University of Guelph, Guelph, ON N1G 2W1 Canada; 2 NMR Centre, University of Guelph, Guelph, ON N1G 2W1 Canada

**Keywords:** *Cannabis sativa* L., Prenylated flavonoids, 6-prenylapigenin, 6-geranylapigenin, UbiA prenyltransferase, Microsomal prostaglandin E synthase-1, Anti-inflammatory, Inflammation, Natural products, Ex planta biosynthesis

## Abstract

**Background:**

*Cannabis sativa* L. accumulates a wide array of specialized compounds, many of which are non-psychotropic and show significant promise in medical and therapeutic applications. One such group of *C. sativa* compounds is prenylated flavonoids, which have emerged as potential treatments for chronic pain and inflammation. Accordingly, the aim of this study was to isolate, identify, and synthesize prenylated flavonoids from *C. sativa* and test their efficacy as anti-inflammatory agents.

**Methods:**

An enriched polyphenol extract from *C. sativa* was fractionated using flash chromatography and high-performance liquid chromatography to isolate prenylated flavonoids. Liquid chromatography–mass spectrometry (LC–MS) and nuclear magnetic resonance (NMR) spectroscopy were employed to determine their structures. Phylogenomic and classical biochemical approaches were combined to identify the enzyme involved in the biosynthesis of the isolated compounds. Finally, these prenylated flavonoids were tested to determine their inhibitory properties against microsomal prostaglandin E synthase-1 (mPGES-1) activity.

**Results:**

Two prenylated flavonoids were isolated from the aerial parts of the *C. sativa* plant using classical chromatographic procedures and identified as 6-prenylapigenin (6-PA) and 6-geranylapigenin (6-GA). A *C. sativa* prenyltransferase (CsPT3) from the UbiA superfamily was identified to complete the final prenylation step in 6-PA and 6-GA biosynthesis from the widespread plant flavonoid known as apigenin. The inhibitory potentials of 6-PA and 6-GA against mPGES-1 activity were approximately as effective as, or better than, that of a leading commercially available inhibitor, MK-886. Molecular docking simulations confirmed strong binding affinities of 6-PA and 6-GA to mPGES-1 compared to its natural substrate.

**Conclusions:**

6-PA and 6-GA are prenylated derivatives of the widespread plant flavonoid known as apigenin. These non-psychotropic flavonoids accumulate in *C. sativa* and exhibit potent inhibition of mPGES-1, a chief mediator in the pro-inflammatory pathway. Identification of the final step in 6-PA and 6-GA biosynthesis, together with their now-established anti-inflammatory activity, presents propitious biotechnological avenues for these therapeutically relevant *C. sativa* compounds.

**Supplementary Information:**

The online version contains supplementary material available at 10.1186/s42238-026-00438-4.

## Introduction

Regarded as one of the world’s oldest cultivated plants, *Cannabis sativa* L. has helped shape the social identity of many traditional societies and has been utilized throughout human history as a raw textile, food source, medicament, and resinous intoxicant (Long et al. 2017; Small 2015). More recently, *C. sativa* and its various derivatives have been lauded for the broad therapeutic outcomes that they afford—anxiety, depression, nausea, sleep disorders, and/or acute and chronic pain are some of the ailments relieved by *C. sativa* consumption (Babson and Bonn-Miller 2014; Blessing et al. 2015; Khalsa et al. 2022; Russo 2016; Whiting et al. 2015).

The phytochemicals from *C. sativa* that offer health-promoting properties include the primary psychoactive cannabinoid, Δ^9^-tetrahydrocannabinol (THC), as well as the various non-intoxicating cannabidiol (CBD), cannabidiolic acid, cannabigerol, cannabichromene, and cannabinol (Leinen et al. 2023; Radwan et al. 2021; Smith 1998). One class of non-cannabinoid constituents from *C. sativa* that have been touted for their therapeutic potential are flavonoids and the related bibenzyls (Abdel-Kader et al. 2023; Boucher et al. 2025; Erridge et al. 2020). Specifically, cannflavins represent a group of prenylated flavonoids that exhibit potent anti-inflammatory activity, neuroprotective effects, and anti-cancer properties (Barrett et al. 1985, 1986; Eggers et al. 2019; Fitzpatrick et al. 2025; Tomko et al. 2022; Werz et al. 2014). Canniprenes include at least three bibenzyl derivatives that inhibit the production of pro-inflammatory eicosanoids and have cytotoxic effects on various cancer cell lines (Allegrone et al. 2017; O’Croinin et al. 2023; Singh et al. 2025).

Relative to other widely distributed and structurally similar phenolic compounds throughout the plant kingdom, the enhanced bioactivity of cannflavins and canniprenes appears to be linked to a key modification—prenylation—of their parent aromatic backbones. Prenylated flavonoids and bibenzyls are rare and are found in only a handful of plant species, typically at very low quantities (Du et al. 2023; Yang et al. 2015). Notably, however, prenylated flavonoids and related aromatics tend to exhibit greater lipophilicity than their non-prenylated counterparts, and it has been postulated that prenylation increases affinity for membranes, resulting in more interactions with membrane-bound targets and/or lipophilic proteins (Brents et al. 2012). Indeed, a wide variety of prenylated aromatics have been investigated for their enhanced therapeutic potential in various disease models (Du et al. 2023; Morante-Carriel et al. 2024; Mukai 2018; Yang et al. 2014).

In planta, prenylation of flavonoids and bibenzyls occurs enzymatically via the transfer of an isoprenoid group to various positions on the flavonoid and/or bibenzyl skeleton (Tello et al. 2008). Ubiquitous allylic diphosphates, dimethylallyl diphosphate (DMAPP) and geranyl diphosphate (GPP), typically serve as the isoprenoid donor in these reactions, and the class of enzymes that catalyze these transfers onto aromatic substrates belongs to the UbiA superfamily of prenyltransferases (Munakata and Yazaki 2024; Tello et al. 2008). The aromatic prenyltransferase family in *C. sativa* is comprised of 12 members (CsPT1–12), which can be conveniently divided into two general groups (Gülck et al. 2020): (1) those that participate in primary processes, such as the biosynthesis of ubiquinone, plastoquinone, and vitamin K (CsPT2, 5, 6, 8, 9, 10, and 11); and (2) those that appear to be involved in the prenylation of “specialized metabolites”, such as the cannabinoids and cannflavins (CsPT1, 3, 4, 7, and 12). This latter group of five enzymes is of particular interest, since they appear to be responsible for the synthesis of several therapeutically relevant prenylated aromatics in *C. sativa* (Luo et al. 2019; Page and Boubakir 2014; Rea et al. 2019).

The potent anti-inflammatory activity associated with *C. sativa* consumption has been, in part, attributed to cannflavins and canniprenes (Bautista et al. 2021). These prenylated aromatics appear to act as dual inhibitors of inflammation by specifically targeting microsomal prostaglandin E synthase-1 (mPGES-1) and 5-lipoxygenase (5-LOX), thereby preventing the synthesis of pro-inflammatory eicosanoids (Allegrone et al. 2017; Barrett et al. 1985, 1986; Werz et al. 2014). As interest in the metabolic diversity of various “strains” of *C. sativa* steadily increases, other prenylated aromatics with similar and/or novel bioactivities are expected to be uncovered. Indeed, a canniprene derivative with cytotoxicity against pancreatic cancer cells was recently identified (Singh et al. 2025) and a prenylated bibenzyl, cannabistilbene I, previously identified by ElSohly et al. (1984), has shown promise in reducing cardiac hypertrophy (Alammari et al. 2024). Accordingly, the goal of this study was to further identify additional prenylated aromatics in *C. sativa* and assess their potential utility as therapeutic agents.

## Methods

### Chemicals and reagents

Apigenin was purchased from B-Thriving, DMAPP was purchased from Echelon Biosciences (I-0051), and GPP was synthesized following the methods outlined by Davisson et al. (1986). Drop-out Mix Synthetic Minus Histidine w/o Yeast Nitrogen Base (D9520) was purchased from USBiological. Dulbecco’s Modified Eagle Medium (11965–092), PBS (10010–023), fetal bovine serum (A52568-01), penicillin–streptomycin (15140122), and Trypsin–EDTA (25300–054) were purchased from Gibco. D-galactose (216310) was purchased from Difco. Bio-Rad protein assay dye reagent concentrate was purchased from Bio-Rad (500–0006). All other chemicals and reagents were purchased from Sigma-Aldrich and Fisher Chemical, unless otherwise stated.

### Isolation and purification of 6-prenylapigenin and 6-gernaylapigenin

A proprietary polyphenol-enriched *C. sativa* extract was supplied by Canurta, Inc. The extract was prepared from the dried aerial parts of two commercially available industrial hemp cultivars, Lindorea and CBG White (< 0.3% THC). Following high-pressure CO_2_ extraction, 10 kg of residual biomass was re-extracted with 85% (v/v) ethanol. The resulting crude extract was subjected to adsorption on a cross-linked, insoluble polyvinylpolypyrrolidone resin, and bound polyphenols were subsequently eluted with ethanol and concentrated. The polyphenol extract was evaporated under reduced pressure at 60 °C, mixed with silica gel (60 Å pore size, 40–63 µm particle size), and fractionated using a flash chromatography system (BUCHI Pure C-810). The sample was separated using Silicycle SiliaSep PREMIUM Flash Cartridges, C18, 25 µm spheres (FLH-03295D-A); using a gradient of water (solvent A) and ethanol (solvent B); increasing from 57% B to 65% B over 10 min; followed by a 13-min isocratic run with 100% B as a post-run wash. Fractions containing 6-prenylapigenin (6-PA) and 6-geranylapigenin (6-GA) were eluted at 5 min and 9 min, respectively. Each fraction was further purified using Hypersep Silica SPE cartridges (5 g bed weight; Thermo Scientific; 60108–711), washed with IPA:hexane (5:95), then eluted with IPA:hexane (50:50). Samples were dried under nitrogen gas and resuspended in methanol.

Each fraction was injected onto an Agilent 1260 Infinity I HPLC equipped with a semi-preparative HPLC Agilent Poroshell 120 EC-C18 column (9.4 mm × 150 mm 2.7 µm; Agilent) column. The fraction containing 6-GA was resolved using solvent A (water, 0.05% formic acid) and solvent B (100% methanol). An isocratic method was employed at 2.8 mL/min and heated at 30 °C, using 80% solvent B over 21 min, causing 6-GA to elute at 18.8 min. This was followed by a 5-min wash period with 100% of solvent B. The fraction containing 6-PA was resolved using solvent A (water, 0.05% formic acid) and solvent B (100% acetonitrile). A 2-min equilibration period using 30% solvent B was employed at a flow rate of 3.0 mL/min and heated at 30 °C, followed by 50% solvent B for 15 min, causing 6-PA to elute at 15.0 min. This was followed by a 6-min wash period with 100% solvent B. The peaks corresponding to these retention times were collected separately using the semi-preparative HPLC method described above and yielded 1 mg each of 6-PA and 6-GA, with purities of ~ 45.5% and ~ 81.7%, respectively (Figure S1). The purified samples were dried (Thermo Savant SPD111 SpeedVac), lyophilized (Labcono Freezone 6 Plus), and then stored at −20 °C until further analysis. Although via gravimetric analysis these compounds proved to be ~ 45.5% and ~ 81.7% pure, spectroscopic analyses (HPLC, MS, NMR) revealed over 90% purity (Figure S1).

### Recombinant expression of CsPT3

The open reading frame of CsPT3 (Figure S2), identical to that used by Rea et al. (2019), was supplied by GenScript. Using PCR, the cDNA was amplified and then ligated between the *Bam*HI and *Xho*I sites of the pESC-HIS plasmid (Agilent). The sequence-verified *CsPT3* plasmid was transformed into *Saccharomyces cerevisiae* (brewer’s yeast) YPH499 (ura3–52 lys2–801^amber^ ade2–101^ochre^ trp1–Δ63 his3–Δ200 leu2–Δ1) according to the methods outlined by Gietz and Schiestl (2007) and Rea et al. (2019). Transformants were selected on 0.013% (w/v) solid synthetic dropout media lacking histidine, containing 0.67% (w/v) yeast nitrogen base and 2% (w/v) dextrose. For protein expression, transformants were cultured as above in liquid selection media until an OD_600_ of 1.0 was achieved. Yeast cells were pelleted via centrifugation, washed twice with sterile water, transferred to liquid selection media containing D-galactose, and then incubated for a further 18 h at 26 °C.

### Prenyltransferase enzyme assays

Transformants expressing CsPT3 were resuspended in 100 mM Tris–HCl, pH 9.0 and then lysed using an EmulsiFlex-C3 (~ 27,000 psi, 10 min). Microsomes were recovered from lysed cells according to the method outlined by Rea et al. (2019). Enzyme assays were conducted with 200 µg of total microsomal protein in a final reaction volume of 200 µL containing 200 µM of apigenin and 400 µM of DMAPP or GPP in 100 mM Tris–HCl + 10 mM MgCl_2_, pH 9.0, and run for 1 h at 37 °C. Inactivated enzymes (boiled microsomes) were used as negative controls in each assay. Reactions were terminated by adding 10 µL of 20% (v/v) formic acid. The substrate and product were extracted through two cycles of phase partitioning (addition of 400 µL of ethyl acetate), dried under nitrogen gas, and resuspended in 60 µL of methanol. A 1 µL injection of each of the extracted enzymatic products was applied to an Infinity Lab Poroshell 120 EC-C18 column (4.6 × 150 mm, 2.7 µm; Agilent) on an Agilent 1260 HPLC Infinity I system; 1 µL of apigenin was injected as a standard. Injections were resolved using a linear gradient of solvent A (45% methanol, 0.1% formic acid) and solvent B (95% methanol, 0.1% formic acid) at a flow rate of 1.25 mL/min and heated at 30 °C. The gradient started at 20% solvent B and increased to 100% solvent B over 8 min, causing 6-PA and 6-GA to elute at 12.3 and 10.3 min, respectively. This was followed by a post-run wash period with 100% solvent B. These compounds were detected by absorbance at 340 nm. Approximately 0.5 mg of 6-PA and 2 mg of 6-GA were collected, dried (Thermo Savant SPD111 SpeedVac), lyophilized (Labcono Freezone 6 Plus), and then stored at −20 °C for further analysis.

### LC–MS analysis

The prenylated flavones isolated from *C. sativa* and the CsPT3 enzyme assay were analyzed using a Waters Aquity i-Class UHPLC system interfaced with a Waters Synapt G2-Si QuanTof™ mass spectrometer. A C18 cartridge column (Agilent Rapid Resolution 2.1 × 30 mm, 3.5 μm) at 30 °C was used with a linear gradient of solvent A (100% water, 0.1% formic acid) and solvent B (100% acetonitrile, 0.1% formic acid) starting at 5% solvent B and increasing to 85% over 18 min. The quadrupole time-of-flight (Q-TOF) mass spectra of both the plant-extracted and enzyme assay-produced isolated compounds matched those of 6-PA ([M + H]^+^ 339.14) and 6-GA ([M + H]^+^ 407.21).

### NMR analysis

Both 1 mg of plant-extracted and 0.5 mg of enzyme assay-produced 6-PA were resuspended in acetone-*d*_*6*_; 1 mg of plant-extracted and 2 mg of enzyme assay-produced 6-GA were resuspended in DMSO-*d*_*6*_ for analysis. Chemical shifts for the plant-extracted compounds were assigned using 1D ^1^H and ^13^C and 2D COSY, HSQC, and HMBC nuclear magnetic resonance (NMR) experiments and compared to the ^1^H chemical shifts of the enzyme assay-produced compounds. NMR spectra were collected on a Bruker AVANCE III 600 MHz spectrometer equipped with a 5 mm TCI cryoprobe. The sample temperature was regulated at 298 ± 1 K. Data were processed in Bruker TopSpin 4.5.0, and spectra were referenced to the residual solvent peaks (Figures S3 and S4).

#### Plant-extracted 6-prenylapigenin

^1^H NMR (Acetone-*d*_*6*_, 600 MHz): δ_H_ = 13.3 (1H, *s*, 5-OH), 7.91 (2H, *d*, J = 8.8 Hz, H-2'/H-6'), 7.03 (2H, *d*, J = 8.8 Hz, H-3'/H-5'), 6.65 (1H, *s*, H-8), 6.62 (1H, *s*, H-3), 5.28 (1H, *tt*, J = 7.3 Hz, 1.4 Hz, H-2"), 3.35 (2H, *d*, J = 7.2 Hz, H-1"), 1.78 (3H, *bs*, H-4"), 1.65 (3H, *bs*, H-5"); ^13^C NMR (Acetone-*d*_*6*_, 150 MHz): δ_C_ = 183.2 (C-4), 164.8 (C-2), 162.5 (C-7), 161.9 (C-4'), 159.9 (C-5), 156.7 (C-9), 131.7 (C-3"), 129.2 (C-2'/C-6'), 123.3 (C-1'), 123.3 (C-2"), 116.9 (C-3'/C-5'), 112.3 (C-6), 105.2 (C-10), 104.2 (C-3), 94.2 (C-8), 26.0 (C-5"), 22.1 (C-1"), 18.0 (C-4").

#### Plant-extracted 6-geranylapigenin

^1^H NMR (DMSO-*d*_*6*_, 600 MHz): δ_H_ = 13.21 (1H, *s*, 5-OH), 10.82 (1H, *s*, 7-OH), 10.33 (1H, *s*, 4'-OH), 7.92 (2H, *d*, J = 8.9 Hz, H-2'/H-6'), 6.92 (2H, *d*, J = 8.9 Hz, H-3'/H-5'), 6.77 (1H, *s*, H-3), 6.53 (1H, *s*, H-8), 5.18 (1H, *t*, J = 7.0 Hz, H-2"), 5.03 (1H, *tt*, J = 7.0 Hz, 1.4 Hz, H-6"), 3.23 (2H, *d*, J = 7.0 Hz, H-1"), 2.00 (2H, *m*, H-5"), 1.91 (2H, *m*, H-4"), 1.73 (3H, *s*, H-10"), 1.58 (3H, *s*, H-8"/H-9"), 1.52 (3H, *s*, H-8"/H-9″); ^13^C NMR (DMSO-*d*_*6*_, 150 MHz): δ_C_ = 181.8 (C-4), 163.5 (C-2), 161.8 (C-7), 161.1 (C-4'), 158.3 (C-5), 155.0 (C-9), 134.2 (C-3″), 130.6 (C-7″), 128.4 (C-2'/C-6'), 124.1 (C-6″), 122.0 (C-2″), 121.2 (C-1'), 115.9 (C-3'/C-5'), 110.9 (C-6), 103.5 (C-10), 102.7 (C-3), 93.2 (C-8), 39.0 (C-4″), 26.1 (C-5″), 25.4 (C-8″/C-9″), 20.9 (C-1″), 17.5 (C-8″/C-9″), 15.9 (C-10″).

#### Enzyme assay-produced 6-prenylapigenin

^1^H NMR (Acetone-*d*_*6*_, 600 MHz): δ_H_ = 13.29 (1H, *s*), 7.91 (2H, *d*, J = 8.8 Hz), 7.03 (2H, *d*, J = 8.8 Hz), 6.67 (1H, *s*), 6.61 (1H, *s*), 5.28 (1H, *t*, J = 7.3, 1.2 Hz), 3.35 (2H, *d*, J = 7.3 Hz), 1.78 (3H, *s*), 1.64 (3H, *s*).

#### Enzyme assay-produced 6-geranylapigenin

^1^H NMR (DMSO-*d*_*6*_, 600 MHz): δ_H_ = 13.20 (1H, *s*), 7.90 (2H, *d*, J = 8.8 Hz), 6.92 (2H, *d*, J = 8.8 Hz), 6.74 (1H, *s*), 6.51 (1H, *s*), 5.18 (1H, *t*, J = 7.3, 1.2 Hz), 5.03 (1H, *tt*, J = 7.0, 1.2 Hz), 3.22 (2H, *d*, J = 7.3 Hz), 2.00 (2H, *m*), 1.91 (2H, *m*), 1.73 (3H, *s*), 1.58 (3H, *s*), 1.52 (3H, *s*).

### mPGES-1 enzyme assays

A549 cells overexpressing mPGES-1 were prepared using the method outlined by Koeberle et al. (2008). In summary, A549 cells (ATCC, CCL-185) were cultured and maintained at 37 °C and 5% CO_2_ in high-glucose (0.45% w/v) Dulbecco’s Modified Eagle Medium (DMEM) with 10% (v/v) heat-inactivated fetal bovine serum, penicillin (50 U/mL), and streptomycin (50 µg/mL). The cells were then trypsinized with 0.05% (w/v) Trypsin–EDTA, quantified, and transferred to 175 cm^2^ flasks (2 × 10^6^ cells grown in 20 mL of culture media), and incubated (16 h). To trigger overexpression of mPGES-1, the culture media was then replaced with fresh high-glucose DMEM supplemented with 2% (v/v) heat-inactivated fetal bovine serum, penicillin (50 U/mL), streptomycin (50 µg/mL), and Interleukin-1β (1 ng/mL) (Invitrogen, A42509) and incubated for an additional 72 h. The cells were then trypsinized with 0.05% (w/v) Trypsin–EDTA, washed with PBS, pelleted, and kept on ice. Then, 0.75 mL of homogenization buffer (100 mM potassium phosphate, pH 7.4, 250 mM sucrose, 2.5 mM glutathione (GSH), 1 mM phenylmethanesulfonyl fluoride, 60 µg/mL soybean trypsin inhibitor, and 1 μg/mL leupeptin) was used to resuspend the pellets. The cells were lysed via sonication on ice (alternating between 20 s of sonication and 20 s of rest) for 2 min at 20 kHz (Fisher Scientific Model 120 Sonic Dismembrator). The cellular lysate was then pelleted via centrifugation (15 min, 4 °C, 10,000 × g). The supernatant was centrifuged again (1 h, 4 °C, 170,000 × g), and 500 µL of homogenization buffer was added to resuspend the microsome-containing pellet. Microsomal protein was quantified using a Bradford assay (Bradford 1976). Microsomes were aliquoted, flash-frozen in liquid nitrogen, and stored at −80 °C for later use. The inhibitory effects of apigenin, plant-extracted 6-PA, plant-extracted 6-GA, and MK-886 (a known inhibitor) on cell-free mPGES-1 enzyme activity were tested in quadruplicate. Each reaction had a total volume of 50 µL and included 20 μg of microsomal protein, 100 μM PGH_2_, and inhibitors at various concentrations (0.5, 1, 5, 10, 20, 40, 80, and 100 μM) in potassium phosphate buffer (100 mM, pH 7.4) containing reduced glutathione (2.5 mM). Reactions were performed for 2 min on ice, then terminated with 50 μL of stop solution (80 mM citric acid and 40 mM iron[II] chloride), followed by centrifugation (20 min, 4 °C, 16,000 × g). Samples were analyzed by injecting 90 μL of the supernatant into an Agilent 1260 HPLC Infinity I system equipped with an InfinityLab Poroshell 120 EC-C18 column (4.6 × 150 mm, 2.7 µm; Agilent) maintained at 40 °C. The sample was resolved using a gradient of solvent A (0.07% aqueous TFA) and solvent B (acetonitrile) at 1.25 mL/min. Solvent B began at 40% and increased to 43% over 8 min, then decreased to 5% over 0.1 min and was maintained at 5% for 2 min. Percent prostaglandin E_2_ (PGE_2_) was detected via absorbance at 195 nm and quantified using a standard curve created using an analytical standard.

### Statistical analysis

A statistical analysis was performed in R, version 4.5.2 (R Core Team 2025; RStudio Team 2022; Wickham 2019) to determine the inhibitory potency of apigenin, 6-prenylapigenin, and 6-geranylapigenin against mPGES-1, relative to MK-886 (a commercially available inhibitor) at various concentrations. We hypothesized that these compounds would exhibit stronger mPGES-1 inhibition than MK-886. A linear regression model was created from the cell-free mPGES-1 activity assay data, in which there was variation of the *y*-intercept and slope of each inhibitor. The percent PGE_2_ formation was analyzed in response to the natural logarithm of each inhibitor’s concentration to ensure a linear relationship. A normal Q–Q plot (Wickham 2016) was generated (Figure S5), and a Durbin–Watson (Fox and Weisberg 2018) test was employed (*p* = 0.468) to ensure that our model satisfied the assumptions of normality of residuals and independence of errors. Likewise, a residuals vs fitted values plot (Wickham 2016) was generated (Figure S6) to confirm that our model satisfied the assumptions of linearity and homoskedasticity. The model’s error terms were determined to fit the following distribution: $i\sim N$(0, 2). The following equation represents the model, where $\beta$_0_ is the *y*-intercept, $\beta$_1_ is the slope, $c$ is the identity of the mPGES-1 inhibitor, $i$ is an individual data point, and $c$[$i$] is the identity of the mPGES-1 inhibitor at the $i$ ^th^ point:

$$Percent\ {PGE}_{2}\ Formation = {\beta }_{0,}{c}_{[\mathrm{i}]}{+{\beta }_{1,}{c}_{[\mathrm{i}]}\mathrm{ln}(\mu M)}_{i}+{\varepsilon }_{i}$$

Informed by the linear regression model described above, one million sets of regression coefficients were simulated (Venables et al. 2002), assuming their sampling distribution approximates a multivariate Gaussian distribution. Each set of simulated regression coefficients was used to compute fitted responses for each flavone. These values were then relativized to the fitted response of MK-886 using the same set of regression coefficients. Simulation-based *p* values were derived from a one-tailed test and reflect the proportion of these ratios exceeding one (exhibiting lower potency MK-886). To visualize the inhibitory difference between these flavones, the median relative response was plotted across all tested concentrations. Error bars represent 90% simulation-based confidence intervals to be consistent with a one-tailed hypothesis test at an alpha level of 0.05.

### Molecular docking of substrates to mPGES-1 in silico

Molecular docking was conducted following the methods outlined by Forli et al. (2016), with an approach similar to that of Lauro et al. (2017). Briefly, the ligands were generated and protonated with Molscrub, then prepared for docking using Meeko. The receptor (mPGES-1) and GSH cofactor structures were obtained using the 4AL0 mPGES-1 crystal structure file from the RCSB Protein Data Bank (RCSB Protein Data 2013). Each asymmetric unit monomer model was combined using UCSF ChimeraX to obtain a single trimer model suitable for docking (Goddard et al. 2018; Meng et al. 2023; Pettersen et al. 2021). BOG, PLM, and solvents were removed from the model using ProDy. The receptor’s “CRYST1” line was preserved, hydrogens were corrected and added using reduce, and the output was recombined with GSH and then prepared for docking using Meeko. Molecular docking was then performed using the AutoDock Vina forcefield with an exhaustiveness of 500 (Eberhardt et al. 2021; Trott and Olson 2010). The *x*, *y*, and *z* coordinates for the docking box were set to 10.304, −11.033, and −8.384, respectively (Lauro et al. 2017). To accommodate the size of 6-PA and 6-GA, the docking box *x*, *y*, and *z* plane dimensions were set to 24, 22, and 32 Å, respectively. Each substrate was simulated with 10 binding configurations, and the configurations with the lowest predicted free energy of binding were chosen for visualization. The protein structure was coloured according to the Eisenberg hydrophobicity scale (Eisenberg et al. 1984), and visualizations were rendered using PyMOL version 3.1.3.1 (Schrödinger, LCC 2015).

## Results

### Purification of prenylated apigenin derivatives from hemp

**Fig. 1:**
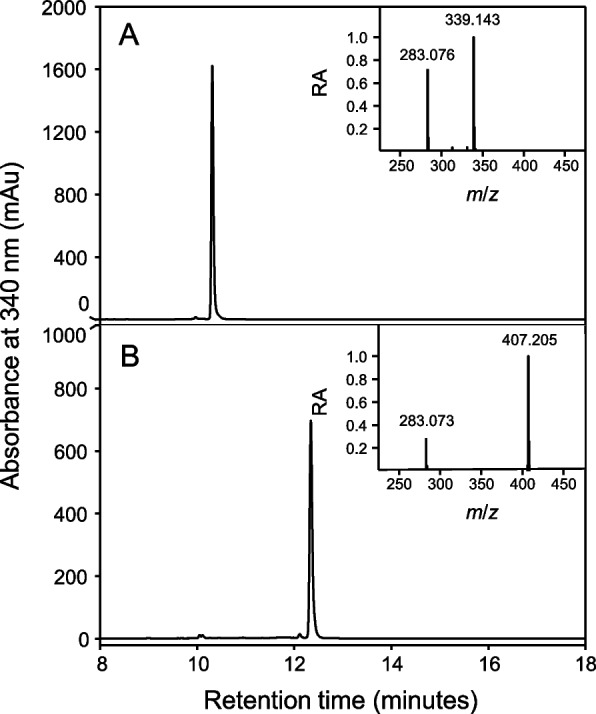
Representative HPLC chromatograms of 6-PA and 6-GA. 6-PA (**A**) and 6-GA (**B**) were isolated from a polyphenol-enriched extract of *C. sativa*. The mass spectra are shown for each compound and depict the mass-to-charge ratios of 6-PA ([M + H]^+^ 339.143) and 6-GA ([M + H]^+^ 407.205). Note the in-source fragmentation ions associated with 6-PA (283.076) and 6-GA (283.073)

As part of an ongoing effort to identify therapeutically relevant aromatic compounds from *C. sativa*, a polyphenol-enriched extract from the aerial parts of two commercially available industrial hemp cultivars (Lindorea and CBG White, each containing < 0.3% THC) was obtained, as described in the Materials and Methods section. From this starting material, we first focused on isolating cannflavins A and B. However, during the isolation process, two minor “contaminants” were consistently observed to elute off of semi-preparative chromatography columns immediately before the cannflavins (Figure S7). The compounds were further analyzed using Q-TOF mass spectrometry, and their mass-to-charge ratios were consistent with being prenylated derivatives of apigenin (a widespread flavone), specifically dimethylallyl apigenin (*m*/*z* 339.14) and geranyl apigenin (*m*/*z* 407.21), as illustrated in Fig. 1. To ascertain the molecular structure and the specific position of the two prenyl groups (dimethylallyl and geranyl) on these apigenin derivatives, NMR analysis was performed (Figures S1 and S2). The assigned ^1^H and ^13^C chemical shifts for dimethylallyl apigenin are in excellent agreement with previous work (Delle Monache et al. 1994; Li et al. 2014) and the assigned ^1^H and ^13^C chemical shifts for geranyl apigenin agree strongly with those previously reported by Kumano et al. (2008). These compounds have been previously reported individually, with dimethylallyl apigenin identified in *Maclura pomifera* (Osage orange) by Delle Monache et al. (1994) and geranyl apigenin (albanin D) in *Morus alba* (white mulberry) by Fukai and Nomura (1991). Most recently, these compounds were also identified as a hemp waste extractive (Marani et al. 2026). We herein refer to these compounds as 6-prenylapigenin (6-PA) and 6-geranylapigenin (6-GA), respectively.

### Biosynthesis of 6-PA and 6-GA by an aromatic prenyltransferase

**Fig. 2:**
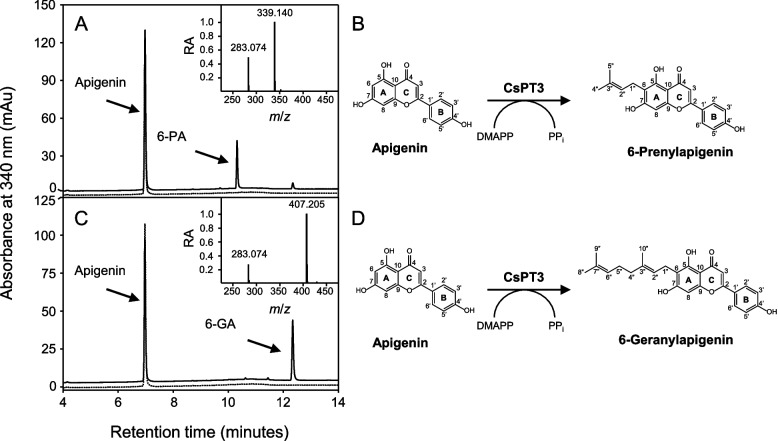
Enzymatic synthesis of 6-PA and 6-GA. Representative HPLC chromatograms and reaction schemes depicting assays with recombinant CsPT3 and apigenin as a substrate along with either DMAPP (**A**, **B**) or GPP (**C**, **D**) as a prenyl donor are illustrated. Note that the synthesis of 6-PA and its associated mass spectrum (**A**, inset) and 6-GA and its associated mass spectrum (**B**, inset) match those of previously isolated 6-PA and 6-GA as indicated in Fig. 1. HPLC chromatograms of assays containing boiled enzyme and the appropriate substrates are indicated by a dashed line

The prenylation of aromatic compounds in plants involves a class of enzymes that belong to the UbiA protein superfamily of prenyltransferases (Munakata and Yazaki 2024; Zhang et al. 2025). *C. sativa* contains a twelve-member family of UbiA prenyltransferases (CsPTs), of which only five are believed to function in the prenylation of “specialized metabolites,” such as cannflavins, canniprenes, and various cannabinoids (Gülck et al. 2020; Page and Boubakir 2012; Rea et al. 2019). We posited that one of these five CsPTs (CsPT1, 3, 4, 7, and 12) is responsible for catalyzing the addition of a dimethylallyl or geranyl group onto position 6 of apigenin’s A-ring, thereby catalyzing the final step in 6-PA and 6-GA biosynthesis. Accordingly, each of these CsPTs was expressed recombinantly in a well-established yeast system and assayed for prenyltransferase activity using apigenin as a substrate and either DMAPP or GPP as prenyl donors. This analysis revealed that only CsPT3 converted apigenin to its corresponding prenylated derivatives using either of the tested cosubstrates, which is consistent with previous observations (Rea et al. 2019). The HPLC retention times, mass-to-charge ratios, and ^1^H NMR assigned chemical shifts of these enzymatic products were consistent with those of the 6-PA and 6-GA that we previously identified (see above), thereby establishing the involvement of CsPT3 in their biosynthesis (Fig. 2). These assays also revealed that CsPT3 converted apigenin into 6-GA approximately 1.8 times more efficiently than into 6-PA, suggesting that GPP is its preferred substrate. These results agree with the kinetic parameters of CsPT3 reported by Rea et al. (2019), where the *Km* for GPP was lower than that of DMAPP.

### Inhibition of mPGES-1 activity by 6-PA and 6-GA

**Fig. 3:**
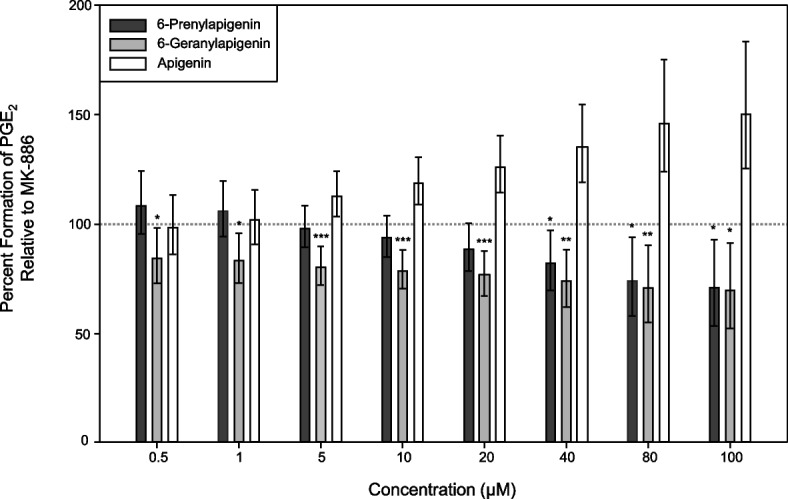
Inhibition of mPGES-1 activity by 6-PA and 6-GA. Microsomes expressing mPGES-1 were isolated from A549 cells and assayed for the conversion of PGH_2_ to PGE_2_. Data show the percent formation of PGE_2_ by mPGES-1 in the presence of apigenin, 6-PA, or 6-GA relative to amount of PGE_2_ formed when mPGES-1 was exposed to the commercially available inhibitor, MK-886 (dashed line). Data are the medians of four independent experiments; error bars depict the 5th and 95th quantiles. Asterisks indicate a significant reduction in PGE_2_ formation compared to MK-886 (*p < 0.05, **p < 0.01, ***p < 0.001)

Several prenylated aromatics from *C. sativa* have been shown to act as anti-inflammatory agents by inhibiting various enzymes in the eicosanoid pathway—for instance, at current, cannflavins A and B, and canniprene have been shown to inhibit mPGES-1, thereby preventing the accumulation of PGE_2_, a key pro-inflammatory mediator (Allegrone et al. 2017; Werz et al. 2014). We therefore performed an in vitro cell-free mPGES-1 activity assay to test the effectiveness of 6-PA and 6-GA as surrogate inhibitors. In parallel, we tested the effectiveness of MK-886, a commercially available and standard inhibitor of mPGES-1 activity (Mancini et al. 2001). In these in vitro assays, both 6-PA and 6-GA demonstrated clear, concentration-dependent inhibition of PGE_2_ production. Across all tested concentrations, 6-GA exhibited significantly greater inhibition than MK-886, whereas 6-PA was approximately equipotent to MK-886, showing significantly greater inhibition only at 40 µM and above (Fig. 3). Notably, apigenin was a poor inhibitor of mPGES-1, suggesting that prenylation critically affects the efficacy of 6-PA and/or 6-GA as bona fide mPGES-1 inhibitors (Fig. 3 and Figure S8). Next, we simulated the binding affinities of apigenin, MK-886, prostaglandin H_2_ (PGH_2_), 6-PA, and 6-GA to the mPGES-1 protein using in silico molecular docking (Figure S9). This analysis revealed strong binding affinities of 6-PA (−8.0 kcal/mol) and 6-GA (−8.6 kcal/mol) to mPGES-1 as compared to apigenin (−7.2 kcal/mol), the MK-886 inhibitor (−6.7 kcal/mol), or its natural substrate, PGH_2_ (−6.3 kcal/mol) (Table 1). Each compound exhibited a docking pose that was positioned on the outer surface of mPGES-1 (relative to its GSH cofactor) at the cytosolic side between subunits of the mPGES-1 homotrimer (Figure S9).

**Table 1:** Molecular docking binding affinities

Compound	Affinity (kcal/mol)^*^
MK-886	−6.7
Prostaglandin H_2_	−6.3
Apigenin	−7.2
6-Prenylapigenin	−8.0
6-Geranylapigenin	−8.6

^*^The in silico predicted free energy of binding values (lower is stronger) of various ligands bound to mPGES-1, calculated using AutoDock Vina (version 1.1.2)

## Discussion

After Fairbairn and Pickens (1981) first reported that THC and CBD-free extracts from *C. sativa* appear to exhibit potent anti-cataleptic properties in mice, a widespread search for non-cannabinoid therapeutics in *C. sativa* began. In this study, we extend these initial observations by describing two prenylated flavonoids from *C. sativa*.

### Prenylated flavonoids in *C. sativa*

To date, the only prenylated flavonoids that have been characterized in *C. sativa* are the cannflavins A, B, and C and isocannflavin B (Bautista et al. 2021). We hereby add 6-PA and 6-GA to this list. Together with canniprenes and cannabistilbenes, this now brings the total number of prenylated aromatic compounds in *C. sativa* to a point that exceeds the number of individual prenyltransferases that are believed to synthesize them. In other words, CsPTs are therefore predicted to accommodate multiple substrates. Indeed, while CsPT3 was originally described to prenylate the widespread flavone known as chrysoeriol into cannflavins A and B, our data indicate that it can also utilize the equally abundant apigenin as a substrate to synthesize 6-PA and 6-GA (Jin et al. 2020; Rea et al. 2019). In both cases, the regiospecificity of CsPT3 for either apigenin or chrysoeriol appears to focus on position 6 of the resorcinol A-ring found on each flavone substrate. Interestingly, this prenylation pattern is not found on any of the other prenylated aromatics in *C. sativa*, which suggests that the synthesis of the canniprenes and cannabistilbenes, for instance, involves another yet-to-be-identified member of the CsPT family.

### Anti-inflammatory potential of 6-PA and 6-GA

A wide range of prenylated flavonoids from plants have previously been shown to exhibit anti-inflammatory properties through various of mechanisms (Lv et al. 2023). For example, 10-oxomornigrol F, a prenylated flavonoid from *Morus alba* (white mulberry), exerts an anti-inflammatory effect by activating the Nrf2/HO pathway via the p38 MAPK pathway (Tran et al. 2018). Xanthohumol from *Humulus lupulus* (common hop) lowers pro-inflammatory markers such as IL-1β, NLRP3 inflammasome, and cleaved caspase-1 in an arthritis mice model (Wang et al. 2023). Icariin and its prenylated derivatives from *Epimedium grandiflorum* (large flowered barrenwort) exhibit significant anti-inflammatory effects by inhibiting the NF-κB signalling pathway and reducing the expression of inducible nitric oxide synthase (Hu et al. 2025).

In this study, we showed that 6-PA and 6-GA inhibit mPGES-1 enzyme activity and thus prevent it from synthesizing PGE_2,_ a key mediator of inflammation. Typically, over-the-counter non-steroidal anti-inflammatory drugs (NSAIDs) are administered to achieve this same effect; however, NSAIDs specifically inhibit cyclooxygenase (COX) enzymes, which are responsible for converting arachidonic acid into PGH_2_, the substrate for mPGES-1 (Bacchi et al. 2012; Rouzer and Marnett 2020). While extremely effective, NSAIDs and other COX-1/2 inhibitors have well-documented side effects, including gastrointestinal toxicity, cardiovascular risks, and renal damage (Fendrick and Greenberg 2009; Minhas et al. 2023; Panchal and Sabina 2023). Because mPGES-1 functions downstream of COX-1/2 and is, in many cases, not required for basic homeostatic PGE_2_ production (which is mediated by cytosolic PGES), it is considered a promising therapeutic target for treating inflammation while minimizing the severe side effects associated with traditional NSAIDs (Ding et al. 2018; Koeberle et al. 2016). This underscores the value of developing future platforms for 6-PA and 6-GA production to capitalize on their therapeutic potential as potent anti-inflammatory agents.

### Mechanistic insight into 6-PA and 6-GA bioactivity

Our in vitro data indicate that 6-PA and 6-GA inhibit mPGES-1 enzyme activity more effectively than a leading commercially available inhibitor, MK-886 (Mancini et al. 2001). The efficacy of these flavones appears to be tied to the presence and size of the prenyl moiety attached to the flavone backbone. Non-prenylated apigenin serves as a poor inhibitor; however, addition of the larger ten-carbon (C10) geranyl group on 6-GA renders this compound a more effective inhibitor of mPGES-1 than 6-PA, which contains a smaller C5 dimethylallyl moiety (Fig. 3; Figure S8). Indeed, our in silico modelling of the mPGES-1 active site (Lauro et al. 2017) indicated that 6-GA exhibited a stronger binding affinity than 6-PA. In this model, apigenin, 6-PA, and 6-GA each adopted similar orientations within the mPGES-1 active site. Prenylation enabled the ligands to occupy a greater proportion of the active site and engage additional hydrophobic regions, potentially explaining the enhanced binding affinities and stronger mPGES-1 inhibition observed for the prenylated compounds (Figure S9 and Table 1). Alternatively, 6-PA and 6-GA could conceivably interact with mPGES-1 allosterically or block entry of the PGH_2_ substrate and/or GSH cofactor to the active site of the enzyme. In this context, cannflavin A was shown to inhibit mPGES-1 activity independently of the PGH_2_ concentration, suggesting a non-competitive mode of inhibition for this structurally-related compound (Werz et al. 2014).

### Concluding remarks

In this study, we have characterized 6-PA and 6-GA as compounds in *C. sativa* that inhibit the in vitro synthesis of pro-inflammatory mediators. As with other natural products with therapeutic potential, these compounds accumulate to very low levels in the plant—our analysis predicts that 6-PA and 6-GA would be present at less than 100 mg/kg in dried plant material, which makes large-scale extraction of the compounds impractical. However, having established that CsPT3 can actively convert apigenin to 6-PA and 6-GA, propitious biotechnology options are now available for their synthesis. Considering that apigenin biosynthesis has already been recapitulated in a yeast system, adding CsPT3 to this strain becomes a rather straightforward exercise to generate 6-PA and 6-GA in significant quantities (Leonard et al. 2005). Alternatively, apigenin can be obtained as a waste material extractive (Bonasia et al. 2023) and can therefore be introduced into immobilized enzyme bioreactors that contain CsPT3 or an engineered version of the protein’s activity (Ni et al. 2020).

## Supplementary Information

Supplementary Material 1.

Supplementary Material 2.

## Data Availability

All data generated or analyzed during this study are included in this published article [and its supplementary information files].

## Abbreviations

6-GA: 6-Geranylapigenin
6-PA: 6-Prenylapigenin
BOG: Octyl beta-D-glucopyranoside
CsPT3: *Cannabis sativa* Prenyltransferase 3
GSH: Glutathione
mPGES-1: Microsomal prostaglandin E synthase-1
PGE_2_: Prostaglandin E_2_
PGH_2_: Prostaglandin H_2_
PLM: Palmitic acid
UbiA: Ubiquinone biosynthesis gene A
