# Supplementary material for: Prenylated apigenin derivatives from Cannabis sativa L.: isolation, biosynthesis, and anti-inflammatory properties
Source: J Cannabis Res. 2026 Apr 16;8:66. doi: 10.1186/s42238-026-00438-4 (PMC13195824; doi:10.1186/s42238-026-00438-4)
Supplement: Supplementary file 1 — Supplementary Material 1. [file 42238_2026_438_MOESM1_ESM.pdf]

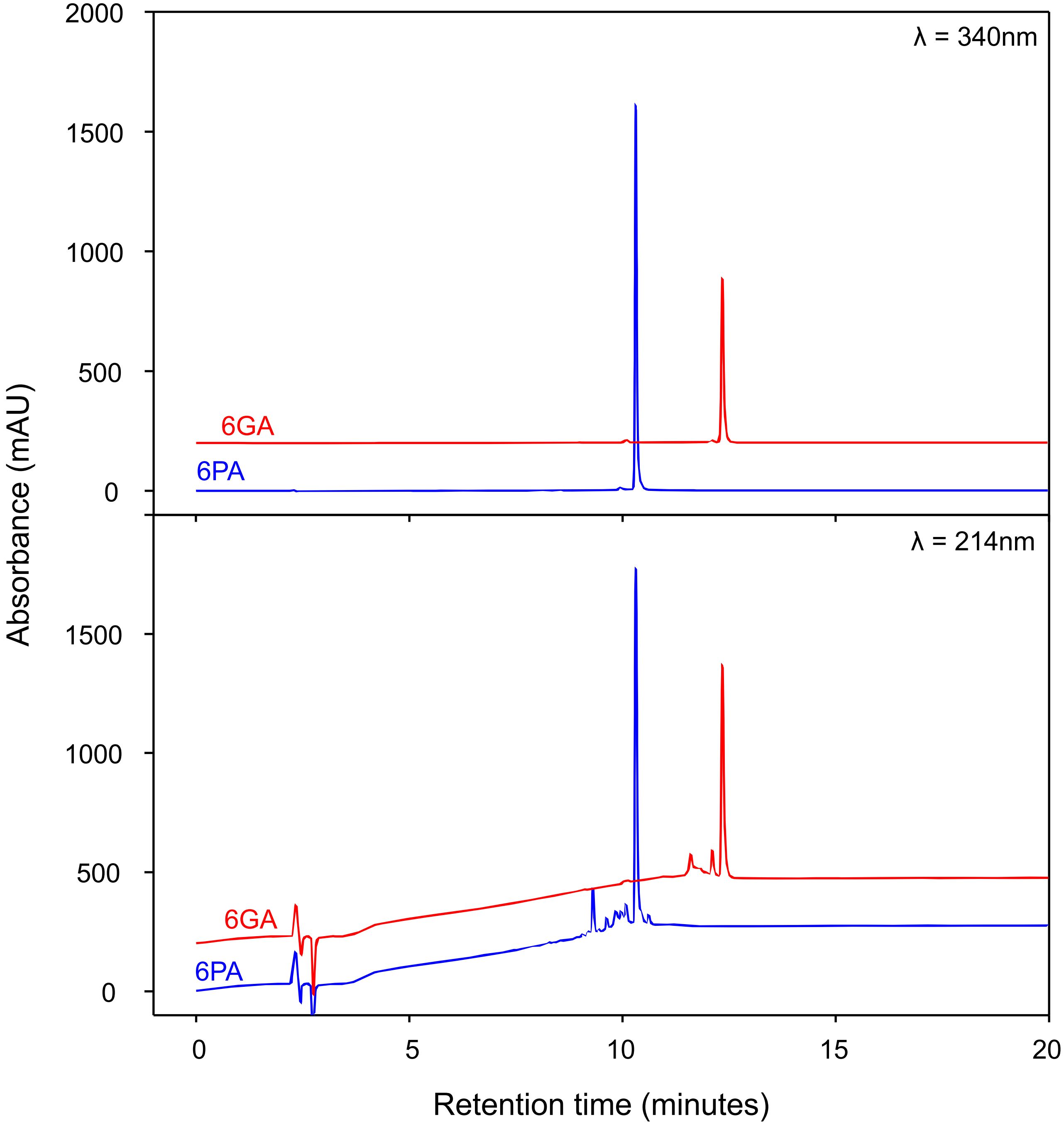

Supplemental Figure 1. Representative HPLC chromatograms of purified 6-PA and 6-GA. Chromatograms were extracted at 340 nm (*upper panel*) and 214 nm (*lower panel*) from purified 6-PA and 6-GA fractions from a polyphenol-enriched *C. sativa* extract.

>ATGGTGTTCTCATCAGTTTGTAGTTTTCCATCCTCCCTTGGAACTAATTTTAAATTAGTTCCTC
GTAGTAATTTTAAGGCATCATCTTCTCATTATCATGAAATAAATAATTTTATTAATAATAAACCAATT
AAATTCTCATATTTTTCTTCAAGACTATATTGCTCTGCCAAACCAATTGTACACAGAGAAAACAA
ATTCACAAAATCATTTTCACTCAGCCACCTCCAAAGGAAAAGCTCCATAAAGGCACATGGTGA
AATTGAAGCTGATGGGAGTAATGGCACATCTGAATTTAATGTAATGAAAAGTGGAAACGCAATT
TGGAGATTTGTAAGGCCATATGCAGCCAAGGGAGTATTGTTTAACTCTGCTGCTATGTTTGCA
AAAGAGTTGGTGGGGAACCTAAATCTATTTAGTTGGCCTTTGATGTTTAAGATACTCTCTTTTA
CATTGGTTATTTTATGCATTTTTGTAAGTACAAGTGGCATCAATCAAATTTATGATCTCGACATC
GACAGGTTAAACAAACCTAATTTGCCAGTAGCATCAGGAGAAATTTCAGTTGAATTGGCATGG
TTGTTGACTATAGTTTGTACAATAAGTGGCCTCACATTAACAATTATAACGAACTCAGGGCCATT
CTTCCCTTTTCTCTACTCTGCTAGTATCTTTTTTGGCTTTCTCTATTCTGCTCCTCCATTCAGAT
GGAAGAAGAATCCTTTTACAGCATGTTTCTGTAATGTTATGTTGTATGTTGGCACAAGCGTTGG
TGTCTATTATGCTTGTAAGGCTAGTCTCGGGCTTCCAGCCAACTGGAGCCCTGCTTTTTGTTT
GCTCTTTTGGTTTATTTCATTGTTGAGTATACCCATCTCCATTGCAAAAGATCTTTCAGACATAG
AAGGTGACCGCAAGTTTGGAATCATAACCTTCTCAACTAAATTTGGAGCAAAACCCATAGCATA
TATTTGTCATGGACTCATGCTTCTGAATTACGTGAGTGTTATGGCTGCAGCTATTATTTGGCCA
CAGTTTTTCAACAGTAGCGTAATATTGCTTTCTCATGCATTCATGGCAATTTGGGTATTATATCA
GGCTTGGATATTGGAGAAATCAAATTACGCCACGGAGACGTGCCAAAAATACTATATATTCCTT
TGGATAATTTTTTCTCTTGAACATGCCTTCTATTTGTTCATGTAG

Supplemental Figure 2. cDNA coding sequence of *CsPT3*.

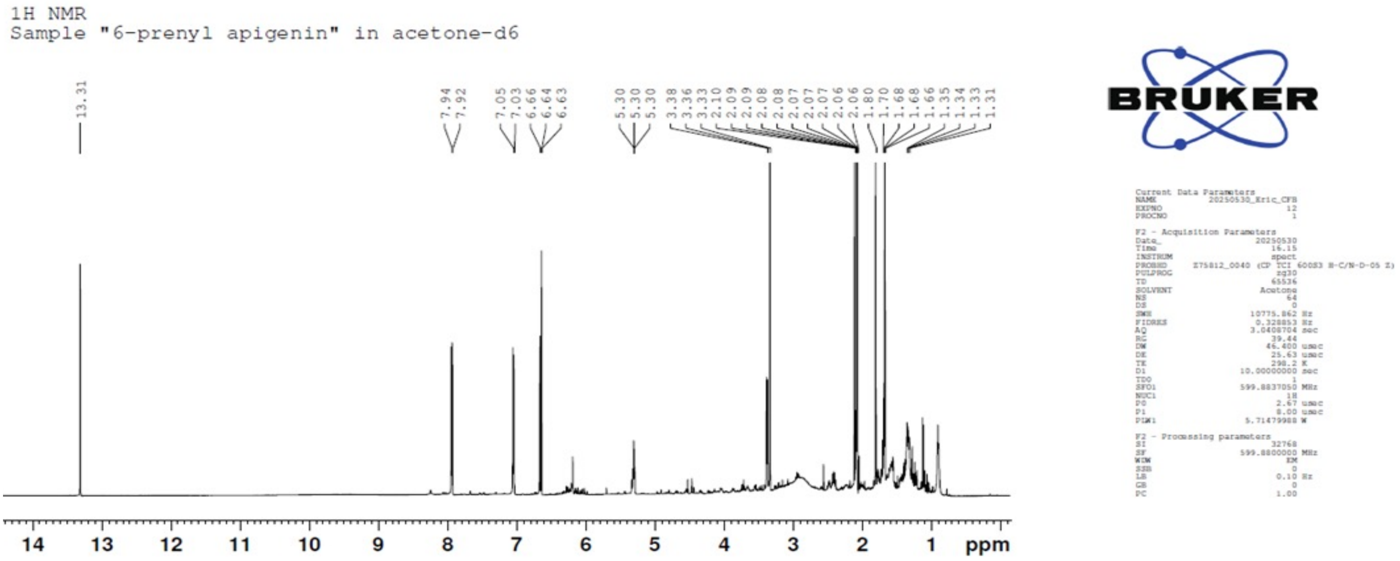

Supplemental Figure 3: The ^{1}H NMR spectrum for compound 1. Compound 1 was extracted from *Cannabis sativa* and analyzed in acetone-d_6 at 600 MHz. Chemical shift assignments are in excellent agreement with those previously reported for 6-prenylapigenin (6-dimethylallylapigenin) (Delle Monache et al. 1994; Li et al. 2014).

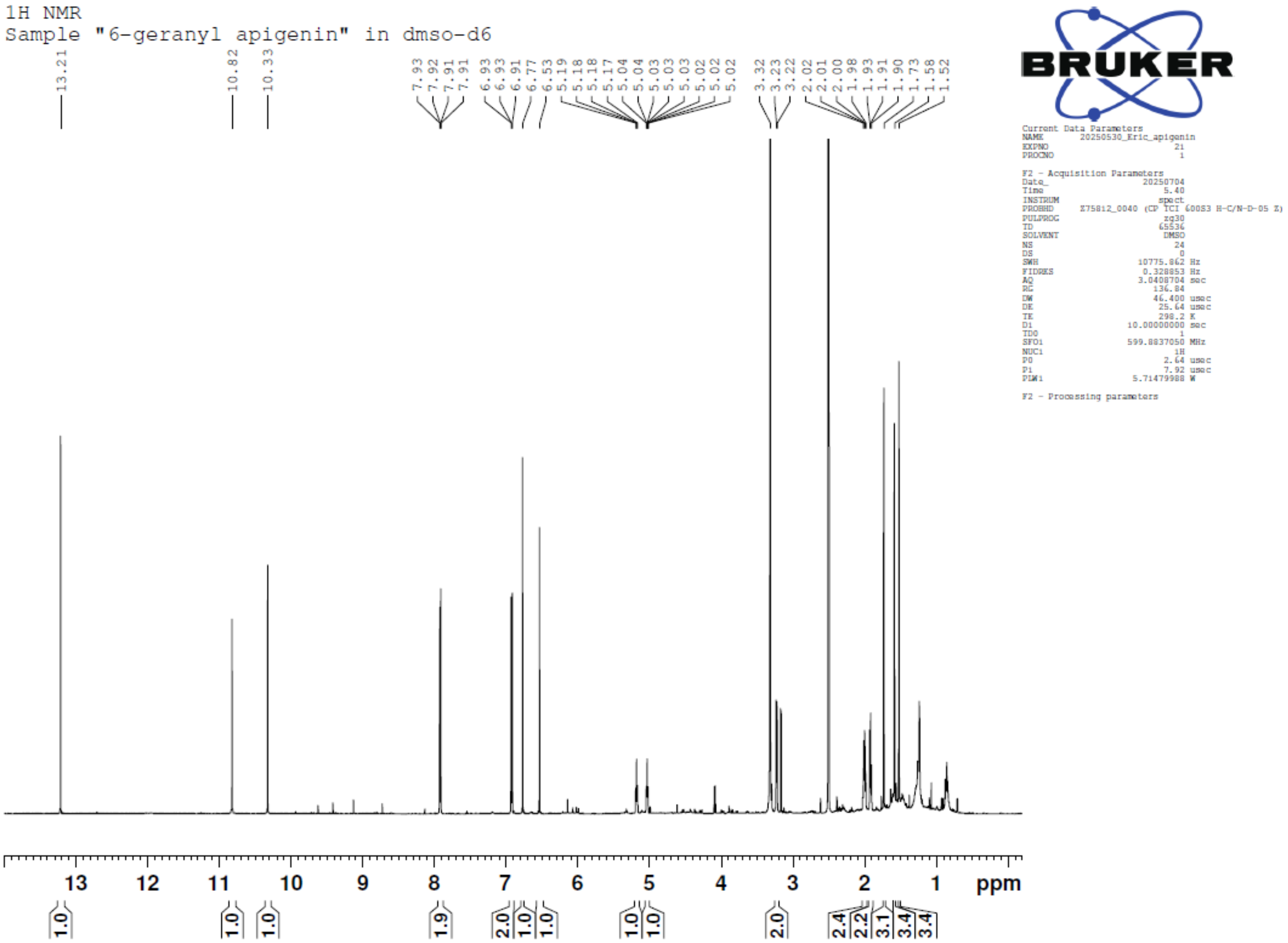

Supplemental Figure 4. The ¹H NMR spectrum for compound 2. Compound 2 extracted from *Cannabis sativa* (analyzed in DMSO-d_6, at 600 MHz). Chemical shift assignments are in excellent agreement with those previously reported for 6-geranylapigenin (Kumano et al. 2008).

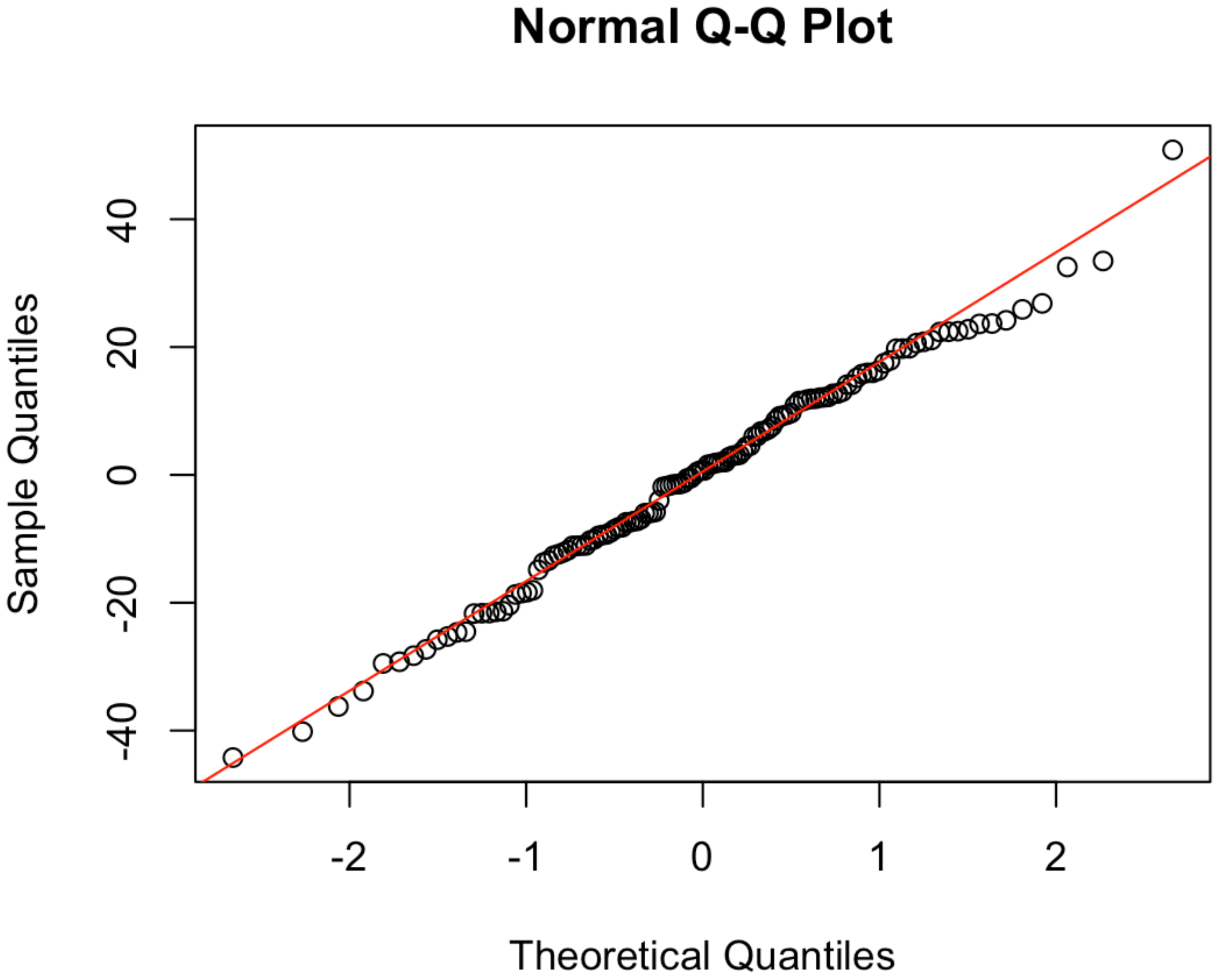

Supplemental Figure 5. A normal Q–Q plot generated from linear regression model. Residuals exhibit a relatively straight line, suggesting the linear regression model used satisfied the assumption of normality of residuals.

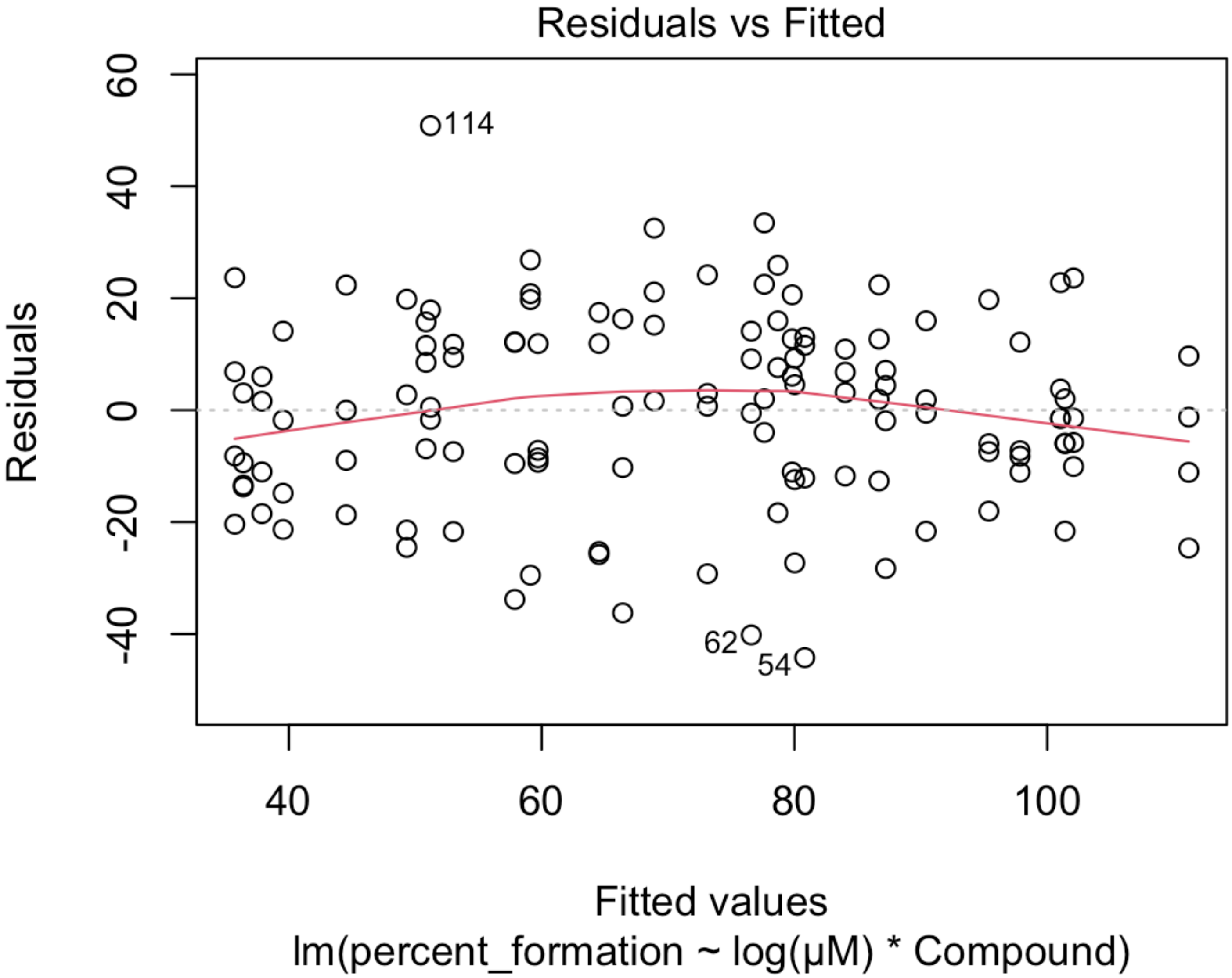

Supplemental Figure 6. Residuals vs fitted values plot. Plot shows the LOESS curve is nearly flat and absent of any strong curve, suggesting the linear regression model used satisfied the assumption of linearity. The plot's vertical spread of residuals is relatively uniform; no sections have an abnormally high variance which therefore satisfies the assumption of homoscedasticity.

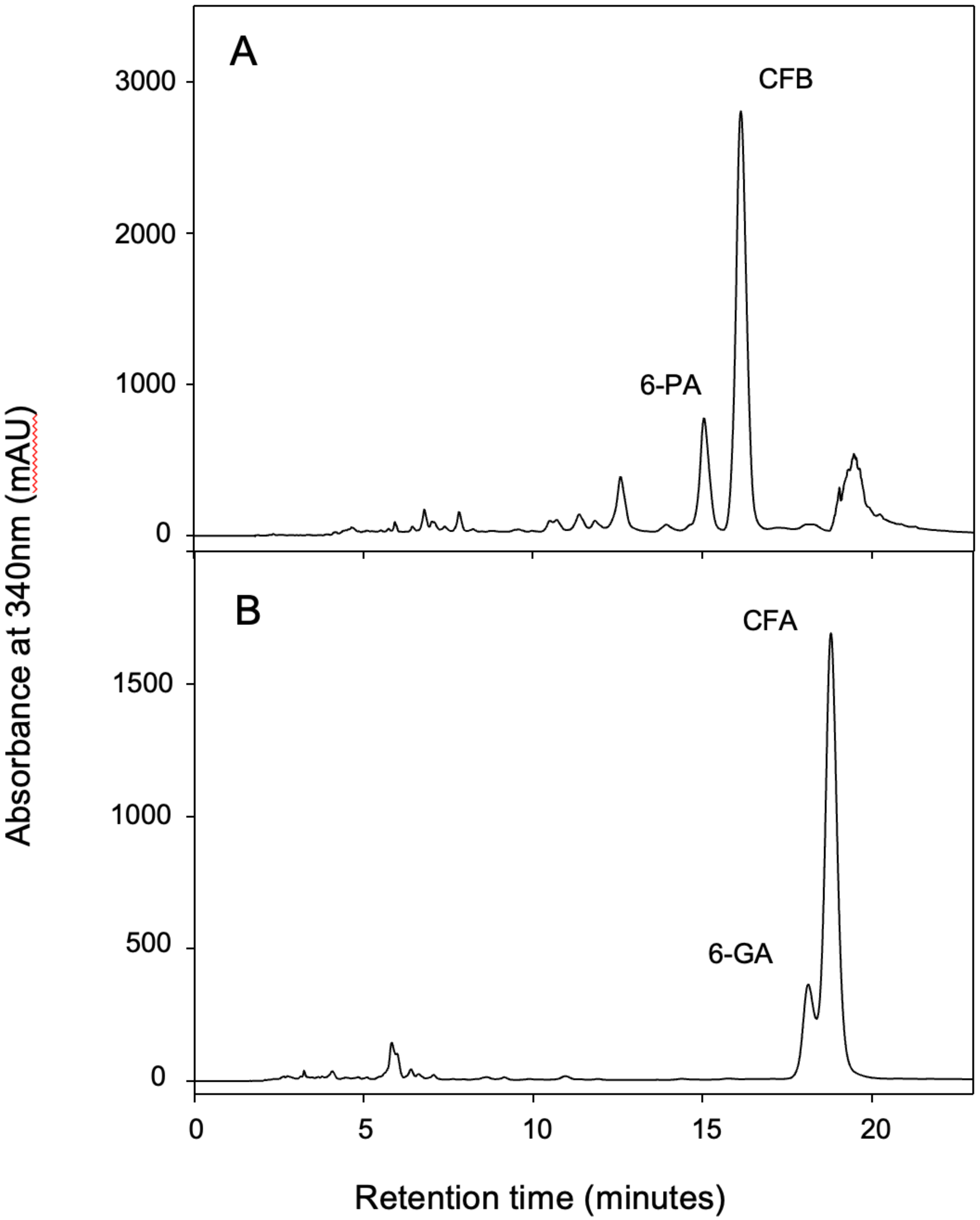

Supplemental Figure 7. Preparative HPLC chromatograms of semi-purified cannflavin B and A extracts. Using the analytical methods described below, compounds 1 **(A)** and 2 **(B)** were consistently observed to elute immediately before the cannflavins, CFA and CFB.

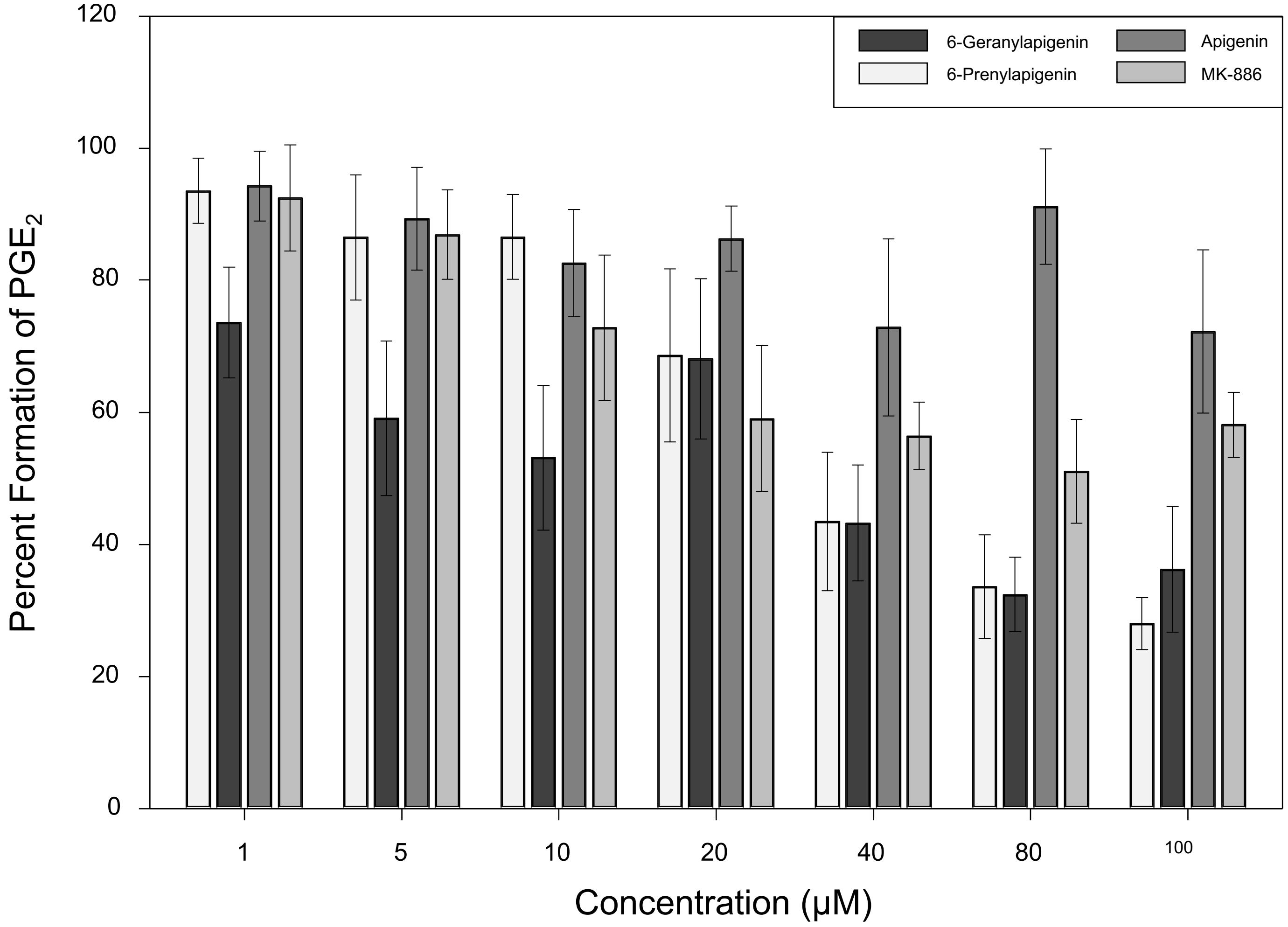

Supplemental Figure 8. Inhibition of mPGES-1 activity by 6-PA and 6-GA. Data show the percent formation of PGE_2 by mPGES-1 in the presence of 6-PA, 6-GA, apigenin, or MK-886. Data are the means of four independent experiments; error bars depict the standard error.

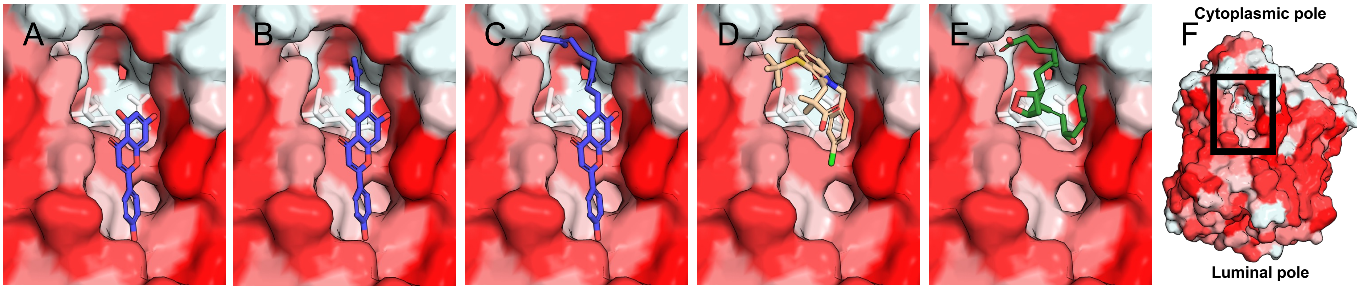

Supplemental Figure 9. In silico docking poses of selected ligands within the mPGES-1 active site. Protein is shown as a surface representation and coloured according to the Eisenberg hydrophobicity scale (red = greater hydrophobicity). The GSH cofactor is shown in white. Ligands are coloured by atom type (C = blue; Cl = lime green; O = red; N = blue; S = yellow). (A) apigenin. (B) 6-PA. (C) 6-GA. (D) MK-886 (beige). (E) prostaglandin H_2 (green). (F) overall structure of the mPGES-1 homotrimer with the ligand active site (black box) and the cytoplasmic and luminal poles indicated. (Refer to the online version of this article to interpret colour references in the figure legend.)

Explanatory Variable	β_0	β_1	Std. error (β_0)	Std. error (β_1)	*p* value (β_0)	*p* value (β_1)
MK-886	95.3796	-9.6681	4.9987	1.6736	$<2\times10^{-16}$	6.12×10^{-8}
Apigenin	2.4687	5.0508	7.0692	2.3668	0.7275	0.0349
6-Prenylapigenin	6.0283	-4.4493	7.0692	2.3668	0.3955	0.0626
6-Geranylapigenin	-15.3570	0.0491	7.0692	2.3668	0.0318	0.9835

Multiple R^2 = 0.6172

Adjusted multiple R^2 = 0.5949

Supplemental Figure 10. Summary Statistics for Linear Regression Model. Regression coefficients (β_0 and β_1) with associated standard errors and *p* values for each explanatory variable (compound). Model fit statistics (multiple R^2 and adjusted multiple R^2 values) are are also reported.
